# Waste PET as a Reactant for Lanthanide MOF Synthesis and Application in Sensing of Picric Acid

**DOI:** 10.3390/polym11122015

**Published:** 2019-12-05

**Authors:** Feng Zhang, Shuyi Chen, Shengqiang Nie, Jun Luo, Shaomin Lin, Yi Wang, Huan Yang

**Affiliations:** 1 College of Chemistry and Pharmaceutical Engineering, Huanghuai University, Zhumadian 463000, China; zhangfenghuaxue@sina.com; 2 Guiyang Inspection Center for Food and Drug Control, Guiyang 550081, China; shuyeechan@126.com; 3 College of Chemistry and Material Engineering, Gui Yang University, Guiyang 550005, China; nieshq1987@163.com (S.N.); 12549033@qq.com (J.L.); 4 School of Material science and Engineering, Han Shan Normal University, Chaozhou 521041, China; lsm678@hstc.edu.cn

**Keywords:** PET, lanthanide metal organic framework, luminescence, sensing of TNP

## Abstract

In this study, a lanthanide metal organic framework based on the ligand of terephthalic acid derived from waste polyethylene terephthalate (PET) bottles was designed and synthesized. The structure and morphology of the Tb-BDC was investigated by X-ray diffractometry (XRD), Fourier transform infrared spectroscopy (FT-IR), and scanning electron microscopy (SEM). The Tb-BDC displays a high selectivity and sensitivity towards picric acid (TNP). The luminescence intensities exhibit a linear relation, with a concentration of TNP over the range of 1 × 10^−5^–1 × 10^−4^ M, with a limit of detection of 1 × 10^−5^ M. The sensing mechanism is also discussed. This is the first time that waste PET materials have been used as the starting precursor of terephthalic acid (BDC) for the fabrication of lanthanide MOF (metal organic framework), which is applied in sensing TNP.

## 1. Introduction

Metal organic frameworks (MOFs), known as crystalline porous architectures, constructed from metal ions and linkers, are fascinating materials which can be applied in various fields [1,2,3,4]. Especially, luminescent lanthanide MOFs have received intense interest owing to their large Stokes′ shifts, high color purity, and long luminescent lifetime [5,6,7]. The combination of inherent luminescence properties, together with the porosity of lanthanide MOFs, is suitable for the sensing of chemicals [8,9,10,11,12,13,14] and temperatures [15,16].

Polyethylene terephthalate (PET) has been largely used in daily life and has raised serious environment issues. The indiscriminate disposal of waste PET causes serious environmental problems by occupying a large volume and PET is quite recalcitrant to degradation [17,18]. The terephthalic acid from PET can be recycled in a number of ways, but few of them are very effective and have high economic costs. The main component of PET is terephthalic acid, which could be hydrolyzed to generate terephthalic acid for MOF synthesis [19]. Terephthalic acid (H_2_BDC) is an attractive ligand due to its rigid structure, its variety of architectures, and its coordination modes on different conditions. Various transition MOFs based on H_2_BDC have been fabricated and have exhibited excellent application properties in different fields. In contrast to the MOFs based on transition metals, the design and fabrication of homologous lanthanide MOFs is in the infant stage [20].

The detection and quantification of low amounts of explosives is a concern for homeland security and environmental pollution. The nitroaromatics, including 2,4,6-trinitrotoluene (TNT), picric acid/2,4,6-trinitrophenol (TNP), and 2,6-dinitrotoluene (2,6-DNT), are used as explosives [21,22]. Among these explosive compounds, high explosiveness and toxicity of TNP can bring negative influence to humans [23]. In spite of these issues, TNP has been widely used in the dye industry and for leather. TNP can easily cause contamination of the soil and ground water [24,25,26]. Although high-explosive detection methods are highly selective and accurate, they suffer from shortcomings including the expense, the need for sophisticated instruments, that they are time-consuming, which limits their use for daily application [27]. There is a need to develop high sensitivity, easily prepared and quick response luminescent sensors for TNP. The luminescent Ln-MOFs and their inherent synthetic versatility seems to be promising for small molecular sensors [28,29].

With the mentioned considerations, combined with our previous work on Ln-MOFs [30,31], herein, a new route is presented to make lanthanide MOF materials from waste PET through a solvothermal method. The obtained Tb-BDC served as a highly selective and sensitive probe for TNP. This is the first time to waste PET had been used as reactant for the preparation of lanthanide MOF, which has applications in sensing TNP. 

## 2. Experimental Section

### 2.1. Materials and Methods

Terbium nitrate was from Sigma Chemical Company. All chemicals were commercially available and used without further purification. 

### 2.2. Instruments

All the photoluminescent properties were tested on a Himadzu RF 5301 PC spectrofluorophotometer (Tokyo, Japan). The UV-Vis spectra of small molecules was performed on a Shimadzu UV 3101PC spectrophotometer (Tokyo, Japan). Fourier transform infrared (FT-IR) spectra were recorded from KBr pellets in the range of 4000–400 cm^−1^ on a Nicolet 330 FT-IR spectrometer (Washington, DC, USA,). Powder X-ray diffraction (PXRD) measurements were carried out using a D/MAX 2200VPC diffractometer with Cu Kα radiation (λ = 1.5406 Å, Tokyo, Japan). The surface morphologies of the material were observed by a scanning electron microscope (SEM, SU8010, Hitachi, Tokyo, Japan). 

### 2.3. Waste PET Hydrolysis

The PET (China YiBao mineral water bottle) was collected from waste bottles and cut into pieces for hydrolysis. The PET pieces were transferred to a Teflon-lined stainless-steel autoclave in different concentrations of NaOH (0.5, 1, 2 mol/L) at 180 °C for 12 and 24 h, respectively, and cooled to room temperature.

The yield of the PET hydrolysis was studied to investigate the effect of sodium hydroxide (20 mL) in different conditions. The results are shown in Table 1.

### 2.4. Synthesis of Lanthanide MOF

A total of 90 mg of BDC (from hydrolyzed PET) was dispersed in 20 mL deionized water. The solution PH value was adjusted to 5 with NaOH (0.5 M), and Tb(NO_3_)_3_·6H_2_O (0.2 mmol, 90 mg) was added to the solution. The final product was washed with DMF (N,N-Dimethylformamide) and deionized water several times, respectively, and filtered off and dried in air. At last, the powder crystalline phase of Tb-BDC was collected.

### 2.5. Photophysical Study

In the luminescent experiment, a 2 mg powder sample (Tb-BDC) was introduced to 4 mL of different small molecules in ethanol and the mixture was shaken for 3 min to form a suspension. Upon excitation at 323 nm, the luminescence response was measured in the 400–700 nm range. By the same method, the emission of Tb-BDC (0.5 mg·mL^−1^) dispersed in different concentrations of TNP (5 × 10^−6^ − 1 × 10^−4^ M) was recorded.

## 3. Results and Discussion

### 3.1. Characterization

**Figure 1:**
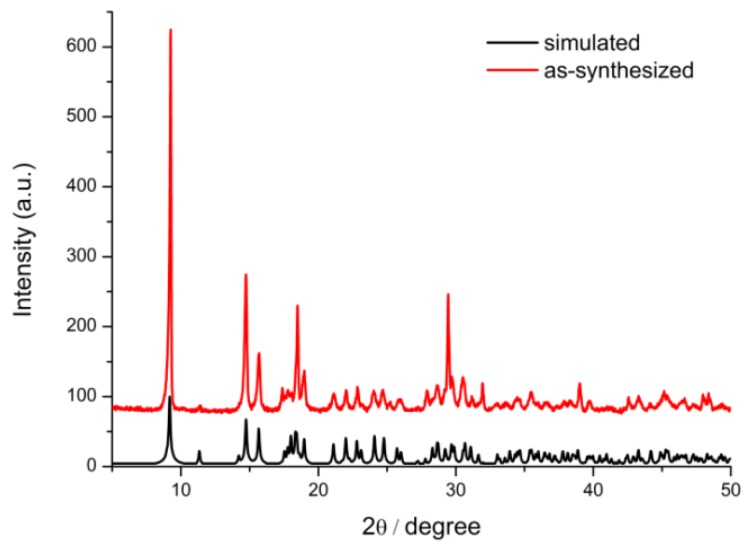
Powder X-ray diffraction (PXRD) spectra of Tb-BDC and simulated Tb-BDC.

Figure 1 presents the PXRD patterns of simulated and as-synthesized Tb-BDC. The PXRD patterns of the Tb-BDC match well with simulated Tb-BDC, suggesting that the material was a successful preparation of simulated Tb-BDC [32]. The structure of (Tb_2_(BDC)_3_(H_2_O)_4_)*_n_* can be described as follows. Tb shows parallelepipedal coordination geometry by coordinating to six oxygens of BDC, in a monodentate fashion, and two water molecules to form an eight-coordinate Tb(III) center. The water molecules occupy the two faces of the parallelepipedal motif. PXRD patterns of simulated and as-prepared H_2_BDC were also performed (Figure S1, Supplementary Materials).

**Figure 2:**
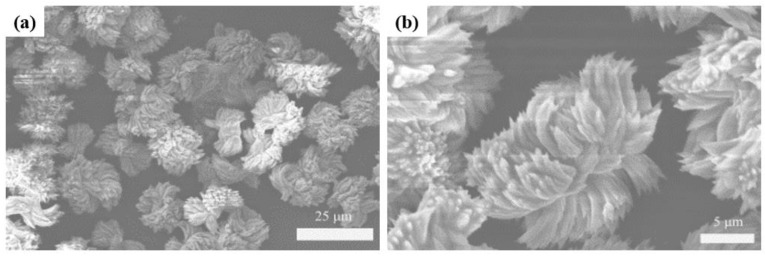
Scanning electron microscopy (SEM) of Tb-BDC: (**a**) Top view; (**b**) enlarged.

Figure 2a,b shows the morphological structure (SEM image) of Tb-BDC. The images show that these flower-like particles, with a size of 10 μm, are dispersed homogeneously. 

**Figure 3:**
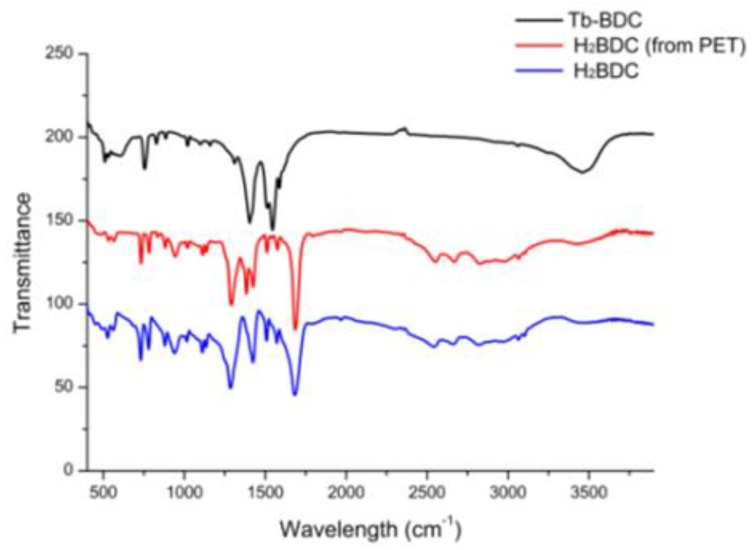
IR spectra of H_2_BDC(PET), H_2_BDC, and Tb-BDC.

The FT-IR spectra of H_2_BDC, H_2_BDC (from hydrolyzed PET), and Tb-BDC are presented in Figure 3. The strong adsorption band at 1691 cm^−1^ and wide adsorption bands of 2500–3000 cm^−1^ in H_2_BDC belong to the –COOH group. In H_2_BDC (PET), all the adsorption bands are consistent with those of H_2_BDC, indicating that PET was successfully hydrolyzed to generate BDC. In the spectra of Tb-BDC, the bands of 1544 and 1403 cm^−1^ are attributed to BDC in the spectra of the Tb-BDC, indicating BDC was successfully coordinated to Tb^3+^.

### 3.2. Photoluminescence Properties

**Figure 4:**
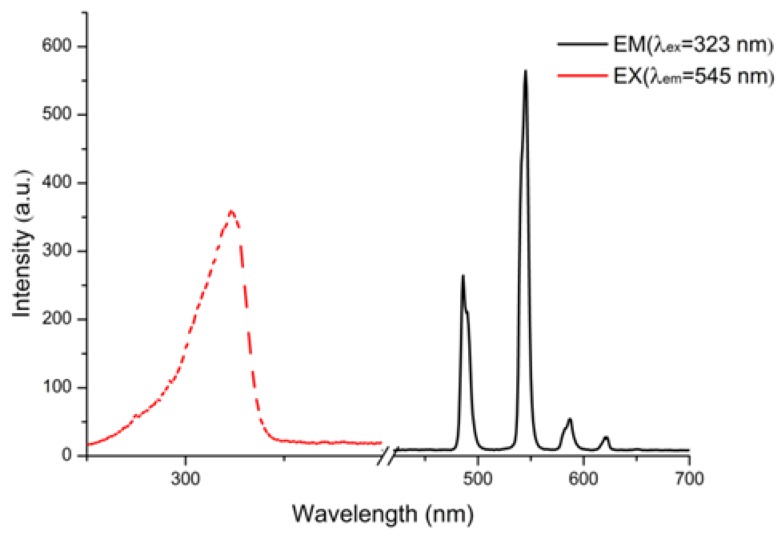
The excitation (dashed) and emission (solid) spectra of the Tb-BDC.

As seen in Figure 4, when excited at 323 nm, Tb-BDC shows four characteristic peaks (^5^D_4_→^7^F_J_ *J* = 6–3) at 489, 545, 592, and 612 nm respectively.

### 3.3. Selective Sensing for Picric Acid

**Figure 5:**
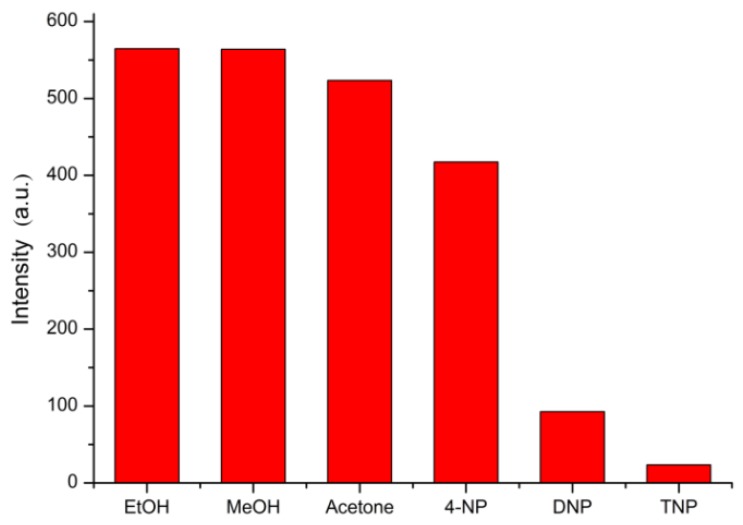
Diagrams of the ^5^D_4_–^7^F_5_ transition intensities of the Tb-BDC at 546 nm in various small organic molecules (λex = 323 nm).

Considering the good luminescence property of Tb-BDC, we investigated the application of Tb-BDC for sensing organic chemicals. The luminescent responses toward a series of small molecules (EtOH, MeOH, acetone, 4-NP, DNP, and TNP) are displayed in Figure 5. Most of the small molecules had a slight change in the luminescence intensity of Tb-BDC at 546 nm, however, the luminescence intensity of Tb-BDC is only quenched in TNP.

**Figure 6:**
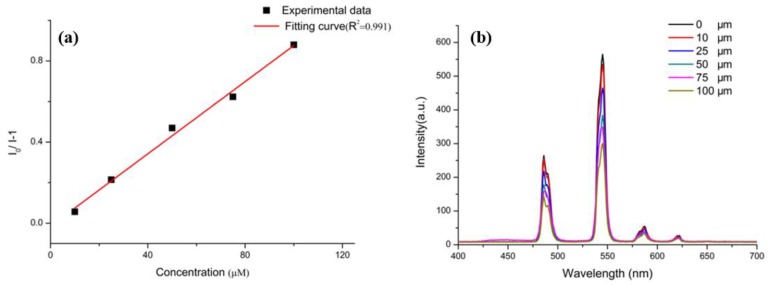
(**a**) Stern–Volmer plot in response to picric acid (TNP); (**b**) Concentration-dependent fluorescence quenching of Tb-BDC upon the addition of different concentrations of TNP.

As shown in Figure 6, the luminescence intensity of Tb-BDC was measured when the content of TNP was different. As the concentration of TNP increased, the intensity of the emission emitted at 545 nm dropped sharply. From Stern–Volmer (S–V) plots we were able to calculate the quenching constants and the quenching efficiency of the analytes using the S–V equation, as follows: (*I*_0_/*I*) = *K*_SV_[A] + 1, where *I*_0_ and *I* are the luminescent intensity in the absence and presence of the analyte, respectively, [A] is analyte concentration, and *K*_SV_ is the quenching constant.[33,34] As a result, the *K*_SV_ of 8 × 10^3^ M^−1^ could be calculated and the limit of detection for TNP could be estimated to be 1 × 10^−5^ M (ΔS/N = 3) (Figure S2, Supplementary Materials) [35,36].

**Figure 7:**
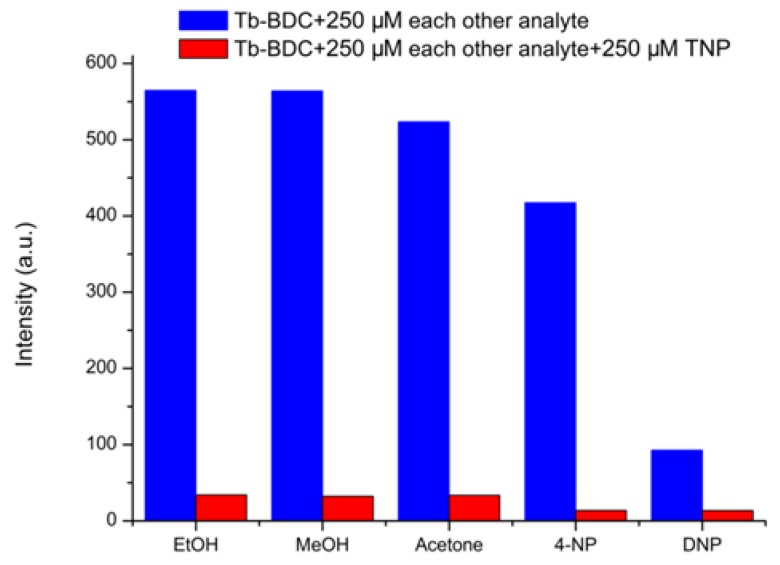
The ^5^D_4_–^7^F_5_ transition intensities of the Tb-BDC upon the addition of different analytes (blue) and subsequent addition of TNP (red) (λex = 299 nm).

Anti-interference ability is one of the indicators for one luminescence sensor. As shown in Figure 7, when the Tb-BDC was used to detect the analytes, such as EtOH, MeOH, acetone, 4-NP, and DNP, the luminescence intensity of the Tb-BDC at 545 nm changed little or was unchanged compared to the original one. However, when commensurate TNP was added to the above analytes (2.5 × 10^−4^ M other analyte + 2.5 × 10^−4^ M TNP), the results show the luminescence was completely quenched, suggesting that the Tb-BDC has high selectivity for TNP.

### 3.4. Luminescent Test Paper

Luminescent test papers were fabricated for demonstrating the practical applications of Tb-BDC through pouring a power suspension of Tb-BDC (0.5 mg/mL) in ethanol onto filter papers, followed by drying, then using them as sensors for the detection of TNP. When the paper was immersed in TNP ethanol solution, the green luminescence of the test paper was completely quenched. The luminescent intensities of the test paper immersed in different small molecules were also performed in the same way (Figure S3, Supplementary Materials). 

**Figure 8:**
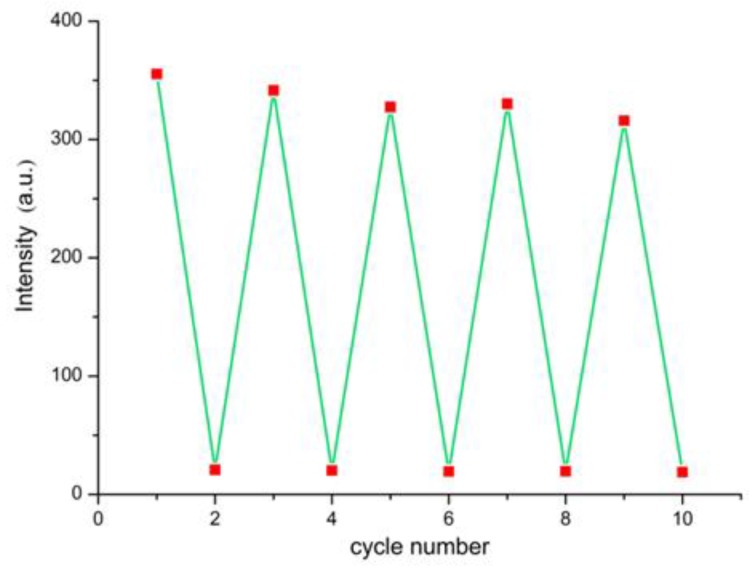
Recyclability of luminescence intensities at 545 nm of the test paper for detecting TNP.

In addition, to investigate the recycle performance, the test paper covered with TNP was handled with water-washing and drying in air. As shown in Figure 8, for sensing TNP, after five washing–drying cycles, the test paper could still recover to 93% of the primitive luminescence intensity.

### 3.5. Quenching Mechanism

**Figure 9:**
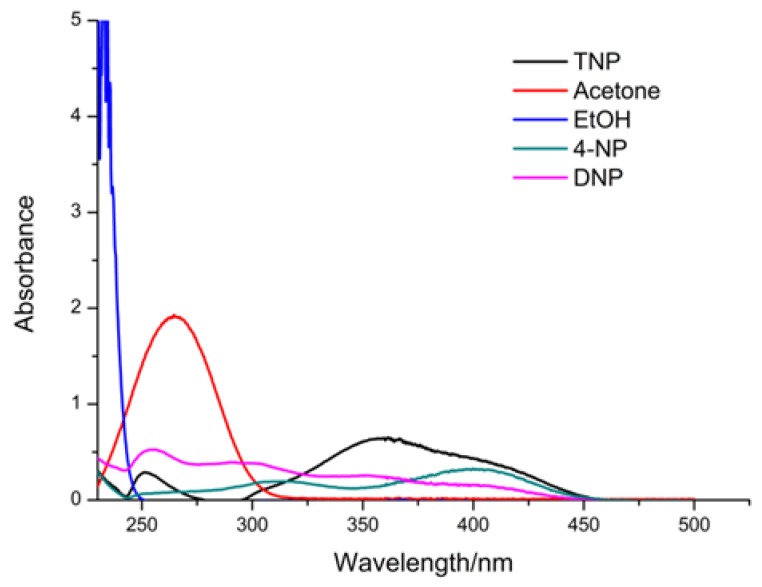
UV absorption spectra of different solvent molecules.

Until now, it was important to investigate the possible quenching mechanism. As shown in Figure 9, UV absorption bands of these small molecules, ranging from 220 to 500 nm, were recorded. 

The UV absorption spectra of TNP reveals that it has a wide and strong absorbance range from 300 to 450 nm. The UV absorption spectra of the other small molecules was also performed. As shown in Figure 9, the absorbing range of TNP shows a higher extent of overlap with the absorption peak of the ligand. This implies that there was a competition of absorption of the light source energy between TNP molecules and the ligand. TNP absorbed most of the energy and only a small fraction of energy was transferred via the ligand to the Tb^3+^ ions. This also illustrates why other small organic molecules had different quenching effects on the Tb-BDC and why the luminescence intensity of the Tb-BDC was decreased or completely quenched upon the addition of TNP.

## 4. Conclusions

In summary, a lanthanide MOF based on the PET bottle material as the resource was synthesized for the first time. The hydrolytic optimum condition for PET was obtained. This is the first time that waste PET bottles have been used as the starting precursor of BDC for the fabrication of lanthanide MOF and for the application of sensing TNP. Although the use of waste PET to produce terephthalic acid and the process for preparing the lanthanide MOF require further research to meet commercial feasibility, the acquired results demonstrate a broader range of environmental protection and effective utilization of waste PET.

## Figures and Tables

**Table 1:** Yields of terephthalic acid (BDC) from polyethylene terephthalate (PET) (China YiBao mineral water bottle, 0.1 g) hydrolysis at 180 ℃ in different concentrations of NaOH and for different times. All hydrolysis experiments were performed in 20 mL deionized water.

NaOH (mol/L)	Crude Product BDC Yield (12 h)	Crude Product BDC Yield (24 h)	Tb_2_(BDC)_3_·(H_2_O)_4_ Yield (24 h)	BDC Yield (24 h)
0	0 g	0 g	0 g	0
0.5	0.02 g	0.07 g	0.054 g	0.031 g
1	0.03 g	0.1 g	0.088 g	0.053 g
2	0.05 g	0.1 g	0.105 g	0.062 g

## Supplementary Materials

The following are available online at https://www.mdpi.com/2073-4360/11/12/2015/s1, Figure S1: Powder X-ray diffraction patterns of smiluated H2BDC and H2BDC (PET); Figure S2: LOD for sensing TNP with the signal of luminescence decrease three times to the noise (ΔS/N = 3); Figure S3: Luminescent response of Tb-BDC coated paper strips to various small molecules under UV light (254nm) (left to right: EtOH, MeOH, acetone, 4-NP, DNP and TNP).
