# Supplementary material for: Waste PET as a Reactant for Lanthanide MOF Synthesis and Application in Sensing of Picric Acid
Source: Polymers (Basel). 2019 Dec 5;11(12):2015. doi: 10.3390/polym11122015 (PMC6960514; doi:10.3390/polym11122015)
Supplement: Supplementary file 1 [file polymers-11-02015-s001.pdf]

Waste PET as a Reactant for Lanthanide MOF Synthesis and Application in Sensing of Picric Acid

Feng Zhang [1,†], Shuyi Chen [2,†] , Shengqiang Nie [3], Jun Luo [3], Shaomin Lin [4], Yi Wang [3,*] and Huan Yang [4,*]

[1] College of Chemistry and Pharmaceutical Engineering, Huanghuai University, Zhumadian 463000, P.R. China
[2] Guiyang Inspection Center for Food and Drug Control, Guiyang 550081, P.R. China
[3] College of Chemistry and Material Engineering, Gui Yang University, Guiyang 550005, P.R. China
[4] School of Material science and Engineering, Han Shan Normal University, Chaozhou 521041, P.R. China
* Correspondence: wy742011@hotmail.com (Y.W.); yanghuan@hstc.edu.cn (H.Y.)
†: The two authors contribute equally to this manuscript.

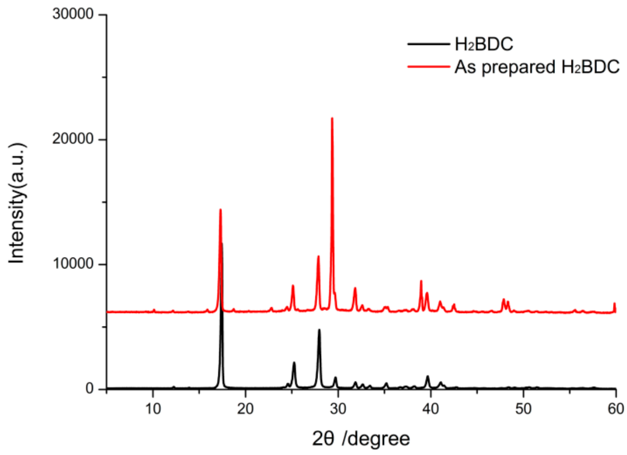

Fig. S1 Powder X-ray diffraction patterns of smiluated H_2BDC and H_2BDC (PET).

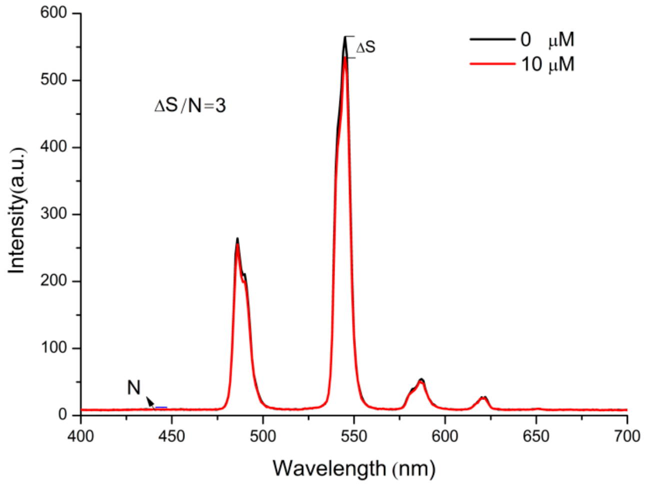

Fig. S2 LOD for sensing TNP with the signal of luminescence decrease three times to the noise (ΔS/N=3)

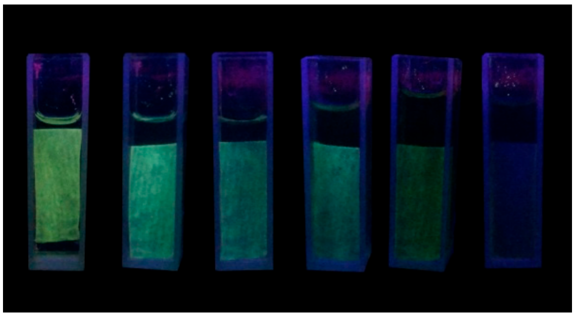

Fig. S3 Luminescent response of Tb-BDC coated paper strips to various small molecules under UV light (254nm) (left to right: EtOH, MeOH, acetone, 4-NP, DNP and TNP).
